# Effects of Huangqi Liuyi Decoction in the Treatment of Diabetic Nephropathy and Tissue Distribution Difference of its Six Active Constituents Between Normal and Diabetic Nephropathy Mouse Models

**DOI:** 10.3389/fphar.2022.934720

**Published:** 2022-06-21

**Authors:** Qun Wang, Ya Shi, Zengguang Wu, Xinli Song, Jinfang Luo, Hong Yang, Xiaolan Chen, Xingde Liu

**Affiliations:** Guizhou University of Traditional Chinese Medicine, Huaxi University Town, Guiyang, China

**Keywords:** huangqi liuyi decoction, pharmacodynamics, HPLC- MS/MS, tissue distribution, active ingredients, nephropathy mouse models

## Abstract

The purpose of this study was to investigate the effects of Huangqi Liuyi decoction extract (HQD) on diabetic nephropathy (DN), and the tissue distribution difference of six main active ingredients of HQD between normal and DN mouse models. DN mice were administered HQD for 12 weeks to investigate its efficacy in the treatment of DN. Liquid chromatography-tandem mass-spectrometry (HPLC-MS/MS) was used to analyze the tissue distribution of the six active ingredients of HQD in normal and DN mice, including astragaloside IV, calycosin-7-O-β-D-glucoside, calycosin glucuronide, ononin, formononetin, and glycyrrhizic acid. DN mice treated with HQD showed significantly decreased fasting blood glucose (FBG), 24-h urinary protein (24 h U-Alb), blood urea nitrogen (BUN), serum creatinine (Scr), and triglyceride levels (TG) (*p <* 0.05). Moreover, there were no significant differences in pharmacodynamics between HQD and Huangqi Liuyi decoction. Treated mice also had decreased expression of collagen I, ɑ–smooth muscle actin (ɑ-SMA), and vimentin; and upregulated expression of E-cadherin in their kidneys. Compared to normal mice, distributions of the six ingredients in the liver, heart, spleen, lungs, kidneys, stomach, small intestine, brain, and muscle of DN mice were different. The results indicated that the HQD could be used for the treatment of DN and to improve renal function. The pathological state of diabetic nephropathy may affect tissue distribution of HQD active ingredients in mice.

## 1 Introduction

Diabetic nephropathy (DN) is an irreversible condition characterized by a continuous decline in the glomerular filtration rate, proteinuria, microalbuminuria, and increased blood pressure (Xiao et al., 2008; Nowak et al., 2018; Kopel et al., 2019). Most individuals with DN progress to end-stage kidney disease (Noor et al., 2020). Prevention or early treatment of DN lowers treatment costs and improves the survival rate and quality of life of patients (Correa-Rotter and Gonzalez-Michaca, 2005). Traditional Chinese medicines (TCMs) have been applied in the clinical treatment of various diseases (Peng et al., 2020). TCMs offer unique advantages in the prevention of diabetic complications because of their limited side effects and/or reduced toxicity (Shi et al., 2011; Sun et al., 2016). Huangqi Liuyi decoction has been used in China since the Song dynasty. It is composed of *Radix Astragali* and *Radix Glycyrrhizae*. *Astragali* inhibits the formation of kidney interstitial fibrosis and retards the development of diabetic nephropathy. *Glycyrrhizae* decreases fasting blood-glucose and kidney oxidative stress (Lu and Wei, 2014; Ma et al., 2019). Huangqi Liuyi decoction significantly decreased fasting blood glucose levels and kidney damage in diabetic rats (Xu et al., 2017; Wen et al., 2018). However, precise and reliable dosing with TCMs remains challenging, which negatively impacts the reproducibility of research and clinical results. Identification of the active constituents of a given compound and ensuring consistency in formulation may overcome the problem with TCM variability (Zhang and Wang, 2005). Relative to DN, our team found that the main active constituents of Huangqi Liuyi decoction were astragalus saponin, astragalus flavone, astragalus polysaccharide, and glycyrrhizic acid (data unpublished). Herein, astragalus saponin, astragalus flavone, astragalus polysaccharide, and glycyrrhizic acid were recombined into mixed extract (HQD), and its effect on DN was determined.

In recent years, ample research has shown that the absorption, tissue distribution, and metabolism of drugs can be affected by the disease state. The pharmacokinetic characteristics in pathological conditions are different from those in the normal condition in a manner directly related to the efficacy and adverse reactions of drugs (Shang et al., 2017; Yang and Liu, 2019; Zhang et al., 2020). Thus, it is necessary to compare the tissue-distribution characteristics of drugs under both normal and pathological conditions. In the present study, a liquid chromatography–tandem mass-spectrometry (HPLC-MS/MS) method was established to investigate differences in the distribution of HQD in tissues and organs under normal and pathological conditions. This study provides additional insights into the safe usage of HQD, and TCMs in general, in healthy mice and those with diabetic nephropathy.

## 2 Materials and Methods

### 2.1 Materials

The reference standards of astragaloside IV (purity >99.0%), calycosin-7-O-β-D-glucoside (purity >98.0%), calycosin glucuronide (purity >98.0%), formononetin (purity >98.0%), ononin (purity >98.0%), and glycyrrhetinic acid (purity >99.0%) were set. Internal standards (IS) were puerarin and digoxin, both with a purity >98.0%, which were obtained from the National Institute for the Control of Pharmaceutical and Biological Products (Beijing, China).

Rosiglitazone (H20052465) was obtained from Shengjitang Pharmaceutical Co., Ltd. (Guizhou, China). Valsartan (H20090319) was obtained from Yijian Pharmaceutical Co., Ltd. (Shangdong, China). Detection kits for 24-h urinary protein (U-Alb) (20180416), serum creatinine (Scr) (20180108), blood urea nitrogen (BUN) (20171215), total cholesterol (TC) (20181005), and triglycerides (TGs) (20171203) were obtained from Nanjing Jiancheng Biological Engineering Research Institute (Nanjing, China). Anti–α–smooth muscle actin (SMA) (BM0002), anti–E cadherin (PB0583), anti–collagen I (BA0325), and anti-vimentin (PB9359) antibodies were obtained from Boster Biological Technology Co., Ltd. (Wuhan, China). A 3,3′-diaminobenzidine immunohistochemistry color-development kit (ZIL-9018) and universal kit (PV-6000) were obtained from Zhongshan Jinqiao Biotechnology Co., Ltd. (Beijing, China).

A mixture of four active constituents (HQD) from Huangqi Liuyi decoction was self-made. In the early stage, the research group determined the preparation process of astragalus saponins, astragalus flavones, astragalus polysaccharides, and glycyrrhizic acid extract. Astragalus saponins and astragalus flavones extract were prepared by macroporus resin column, astragalus polysaccharides extract was prepared by water extraction and alcohol precipitation, and glycyrrhizic acid extract was prepared by acid precipitation. The extract content of each component extracted from three batches of *Astragalus* and *Glycyrrhiza* is shown in Table 1.

**TABLE 1:** The content of each component (x±SD, *n* = 3).

Extract	Component	Content (%)
Astragalus saponins extract	Astragalus saponins	72.97 ± 1.06
	Astragaloside IV	2.72 ± 0.10
Astragalus flavones extract	Astragalus flavones	70.58 ± 2.16
	Calycosin-7-O-β-D-glucoside	1.67 ± 0.08
	Calycosin-glucuronide	1.45 ± 0.10
	Ononin	0.91 ± 0.09
	Formononetin	0.32 ± 0.02
Astragalus polysaccharides extract		67.12 ± 2.60
Glycyrrhizic acid extract		81.02 ± 1.04

According to the ratio of Huagqi Liuyi decoction (*Astragalus*:*Glycyrrhiza*, 6:1), we used 18 kg of *Astragalus* and 3 kg of Glycyrrhiza, through processing, to obtain 121.59 g of astragalus saponins (73.92%, including 2.65% of astragaloside IV), 40.72 g of astragalus flavone (68.93%, including 1.71% of calycosin-7-O-β-D-glucoside, 1.53% of calycosin-glucuronide, 0.93% of ononin, and 0.31% of formononetin), 262.52 g of astragalus polysaccharides (68.67%), and 31.72 g of glycyrrhetinic acid (81.09%). By mixing astragalus saponins, astragalus flavones, astragalus polysaccharides dry extract, and glycyrrhizin extract together, the HQD samples needed for this experiment were obtained.

### 2.2 Animals

Db/db mice can develop nephropathy at 12 weeks of age (Gerald, 2013; Ponchiardi et al., 2013). Ten-week-old db/db male mice and db/m mice were obtained from the Model Animal Research Center of Nanjing University [qualified no. SCXK (Su) 2018–0012] and raised for 2 weeks in a specific-pathogen free laboratory (SPF) at the Experimental Animal Center of Guizhou University of Traditional Chinese Medicine. All mice were housed in polypropylene cages and maintained under standard conditions (25 ± 20°C; relative humidity, 60% ± 5%). Animal studies complied with the European Community guidelines (EEC Directive of 1986; 86/609/EEC) and were approved by the Animal Ethical Committee of Guizhou University of Traditional Chinese Medicine (NO1902137).

### 2.3 Effect of Huangqi Liuyi Decoction on Diabetic Nephropathy

#### 2.3.1 Determination of Biochemical Indexes

Male db/db mice aged 12 weeks were randomly divided into 6 groups of 6 mice each. Twelve-week-old db/m mice served as a control group. The clinical crude drug dosage of Huangqi Liuyi decoction was *Astragalus* 60 g/d and *Glycyrrhiza* 10 g/d, with a total dose of 70 g/d (Xv et al., 2017). *Astragalus* and *Glycyrrhiza* were decocted 3 times, the extract was concentrated and dried, and the dosage of db/db mice was converted according to the above clinical dosage of the drug. The dosage of Huangqi Liuyi decoction was 10.62 g/kg. Mice treated with rosiglitazone (0.61 mg/kg) and valsartan (12.13 mg/kg) served as a positive control group. The doses of HQD in high-, medium-, and low-dose groups were 0.96 g/kg, 0.48 g/kg, and 0.24 g/kg, respectively, which were 4 times, 2 times, and 1 times the clinical dosage. Each group was treated daily for 12 weeks. The control group was given the same volume of distilled water. All mice were fasted for 12 h before the experiment, the FBG level was measured. In addition, 24-h urine was collected, and the 24-h U-Alb level was determined using a Coomassie brilliant blue quantitative method (Nie et al., 2019). The BUN, Scr, TG, and TC values were determined using a standard biochemical apparatus (Roche, cobacc501, Switzerland). Subsequently, the mice were humanely euthanized and their kidneys harvested.

#### 2.3.2 Histopathology Analysis of Kidney Tissues

Paraffin-embedded kidney specimens were sectioned into slices with a thickness of 3 μm using a microtome. After deparaffinization and rehydration, the slices were stained with hematoxylin and eosin for the assessment of kidney injury and with Masson’s trichrome staining for a collagen-deposition assessment. The slices were observed under a light microscope, and images were captured at a magnification of ×400 using the objective lens of an Olympus microscope (Olympus Corporation, Tokyo, Japan). The light microscopy evaluation was conducted by experienced pathologists in a blinded fashion.

#### 2.3.3 Immunohistochemical Assay

Kidney tissue sections (3-μm thickness) were deparaffinized with xylene and rehydrated in a gradient ethanol finishing in phosphate-buffered saline. Endogenous peroxidases were quenched by a few drops of H_2_O_2_. A citrate buffer solution was used to restore the antigens, and the kidney tissues were then sealed with goat serum. After the sections were incubated with the primary antibody overnight, the secondary antibody was applied. Then, the sections were washed with phosphate-buffered saline, dminobenzidine (DBA) was added for color rendering, and counterstaining was completed with hematoxylin. A positive expression was indicated by a brownish-yellow color. For immunohistochemical staining, the average integrated positive area from 6 randomly chosen regions was calculated by using Image Pro Plus 6.0 image analysis software.

#### 2.3.4 Statistics

All data are presented as mean ± standard deviation values. Statistical analysis between the 2 groups was performed using the Statistical Package for the Social Sciences version 23 software program (IBM Corporation, Armonk, NY, United States). *p* ≤ 0.01 and *p* ≤ 0.05 between the 2 groups were considered statistically different.

### 2.4 Tissue Distribution of Huangqi Liuyi Decoction in Control and Diabetic Nephropathy Mice

#### 2.4.1 Preparation of Standard Samples

Stock solutions were separately prepared by dissolving astragaloside IV (5.34 mg), calycosin-7-O-β-D-glucoside (5.05 mg), calycosin glucuronide (5.19 mg), ononin (5.07 mg), formononetin (5.23 mg), glycyrrhizic acid (5.09 mg), puerarin (IS, 5.08 mg), and digoxin (IS, 5.02 mg) in methanol to yield the following concentrations: astragaloside IV, 0.534 mg/ml; calycosin-7-O-β-D-glucoside, 0.505 mg/ml; calycosin glucuronide, 0.519 mg/ml; ononin, 0.507 mg/ml; formononetin, 0.523 mg/ml; glycyrrhizic acid, 0.509 mg/ml; puerarin, 0.508 mg/ml; and digoxin, 0.502 mg/ml. All solutions were stored at 4°C.

#### 2.4.2 Tissue Sample Preparation

Tissues were accurately weighed and homogenized 4 times with normal saline. Then, 200 μl of tissue homogenate and 20 μl of IS solution (60.9 ng/ml of puerarin and 4.02 μg/ml of digoxin) were added into 400 μl of methanol–acetic acid (40:1, v/v). Samples were vortexed for 2 min and centrifuged at 6,000 r/min for 10 min. The supernatant was transferred and evaporated to dryness with a nitrogen-blowing instrument (Organomation, Berlin, MA, United States) at 37°C, and the residue was sonicated with 200 μl of 50% methanol and centrifuged for 10 min at 10,000 rpm. One microliter of supernatant was injected into the HPLC-MS/MS system for analysis. Quality control samples were prepared separately in the same way.

#### 2.4.3 Conditions of HPLC-MS/MS

**FIGURE 1:**
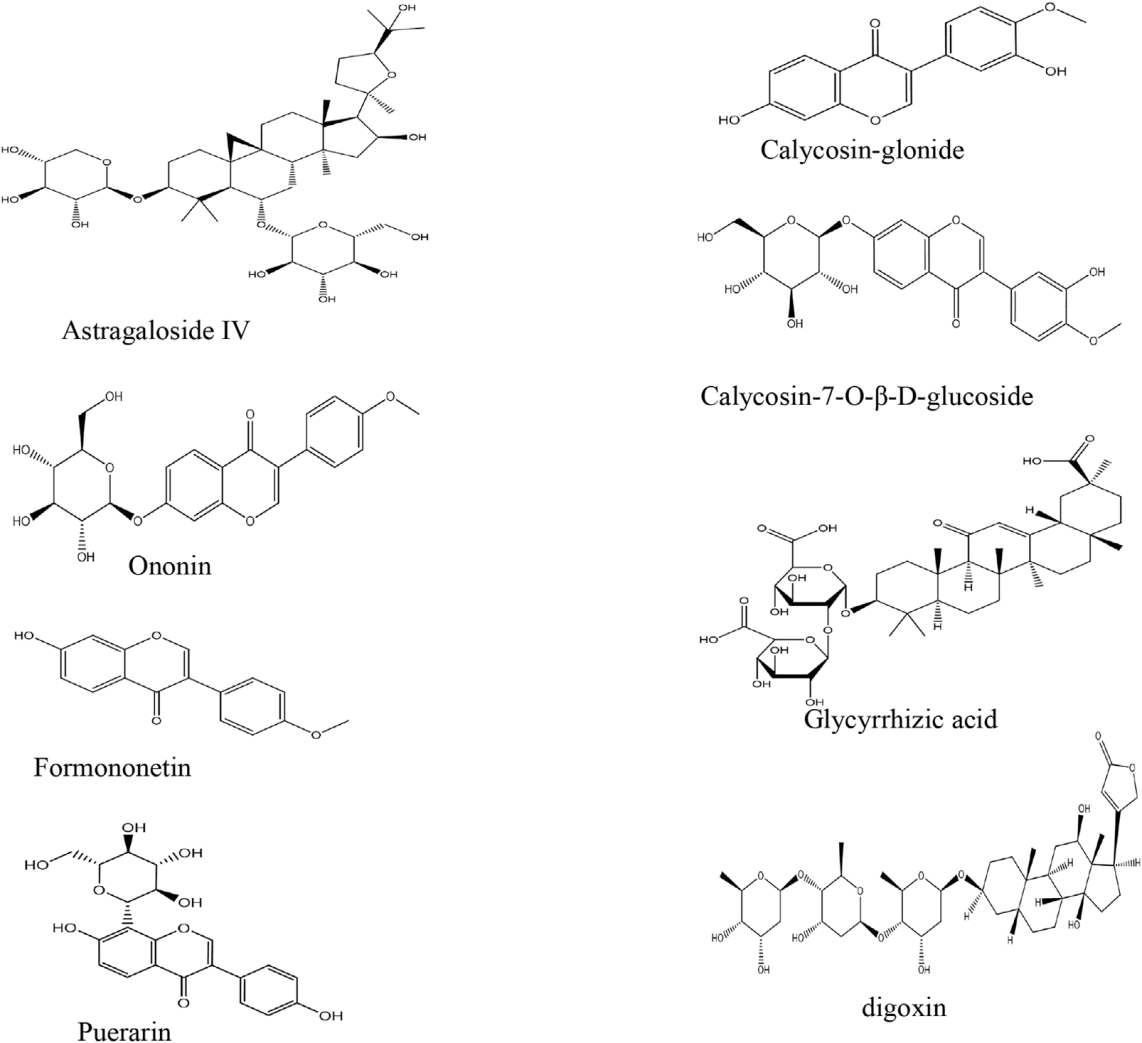
The chemical structure of six analytes.

An Acquity HPLC system (Shimadzu Corp., Kyoto, Japan) equipped with a Q-Trap® 5500 triple quadrupole mass spectrometer (AB Sciex, Framingham, MA, United States) was employed for HPLC-MS/MS. The chromatographic conditions of the six constituents of HQD were determined using an Excel2C18-AR system (100 × 2.1 mm, 2 μm; Advanced Chromatography Technologies Ltd., Aberdeen, Scotland) maintained at 30°C. Analysis was completed with a gradient elution of 0.1% formic acid (A) and acetonitrile (B) and a flow rate of 0.4 ml/min. The gradient elution was as follows: 0–0.6 min (90% A), 0.6–2 min (90%→70% A), 2–6 min (70%→35% A), 6–8 min (35%→10% A), 8–9 min (10%→10% A), 9–9.1 min (10%→90% A), and 9.1–12 min (90% A). For MS/MS detection, an electrospray ionization in a multi-reaction monitoring mode was operated with polarity switching between negative and positive ion modes. The mass spectrometer parameters were set as follows: ion spray voltage at 5.5 kV (+) and −4.5 kV (−), source temperature at 600°C, nebulizer pressure at 55 psi, curtain gas at 30 psi, and auxiliary gas at 55 psi. The multiple reaction monitoring (MRM) analysis was conducted by monitoring the precursor ion to product ion transitions of *m/z* 807.4→627.4 for astragaloside IV, 447.1→285.2 for calycosin-7-O-β-D-glucoside, 285.3→213.2 for calycosin glucuronide, 267.0→252.0 for formononetin, 431.3→269.1 for ononin, 824.4→309.4 for glycyrrhizic acid, 417.1→267.1 for puerarin, and 825.3→649.5 for digoxin. Figure 1 shows the chemical structure of six analytes.

#### 2.4.4 Method Validation

##### 2.4.4.1 Specificity

The specificity of the method was evaluated by analyzing homogenates of drug-free tissue, homogenates of drug-free tissue containing standard solutions and IS, and homogenates obtained following oral administration of HQD to check whether the determination was interfered with by endogenous substances.

##### 2.4.4.2 Calibration Curves and Linearity

The linearity of the calibration curve was constructed using eight calibration points for tissue homogenate. Briefly, peak area analyte/IS ratios (Y) were tested against the theoretical concentration of each analyte (X) using 1/X^2^ weighting of the linear regression. The lowest concentration in the calibration curve was defined as the lower limit of quantification.

##### 2.4.4.3 Accuracy and Precision

The quality control samples at three concentration levels of analytes were prepared and operated in parallel according to the above methods of sample preparation. Each concentration was analyzed during six replications. Assay precision was calculated using relative standard deviation (RSD, %) and variance, and accuracy is expressed as mean ± standard deviation.

##### 2.4.4.4 Extraction Efficiency and Matrix Effect

The recovery and matrix effect of the analytes were analyzed for medium concentration quality control samples. The extraction recovery was determined by comparing the response ratio of extracted samples with extracted blank matrix spiked with corresponding concentrations. The matrix effect was evaluated as the peak area ratio of analytes spiked with blank tissue extract at medium concentrations to non-extracted QC standard solutions at equivalent concentrations.

##### 2.4.4.5 Stability

Quality control samples at three concentration levels of tissue samples were prepared to investigate the stability of tissue samples. First, we stored samples at room temperature (approximately 25°C) for 24 h, and then froze (−20°C) them for 48 h and repeated this freezing and thawing cycle three times. The samples were processed based on the above-mentioned sample processing method and then measured by HPLC-MS/MS.

#### 2.4.5 Tissue Distribution

Thirty-two db/db model mice and 32 normal db/m mice were fasted for 12 h. Then, the diabetic and normal mice were randomly divided into 4 groups of 8 mice each. One group of mice served as a unique sampling time point. Mice were given a single dose of HQD (high dosage in efficacy study). Blood was collected by retro-orbital venous puncture at 30 min and 2, 4, and 8 h. Subsequently, the mice were humanely euthanized, and the heart, liver, spleen, lungs, kidneys, stomach, small intestine, brain, and skeletal muscle were collected. Samples were washed with normal saline and stored at −20°C. Before the experiments, the tissues were prepared according to the sample-preparation methods and analyzed by HPLC-MS/MS.

## 3 Results

### 3.1 Effect of Huangqi Liuyi Decoction on Diabetic Nephropathy

**FIGURE 2:**
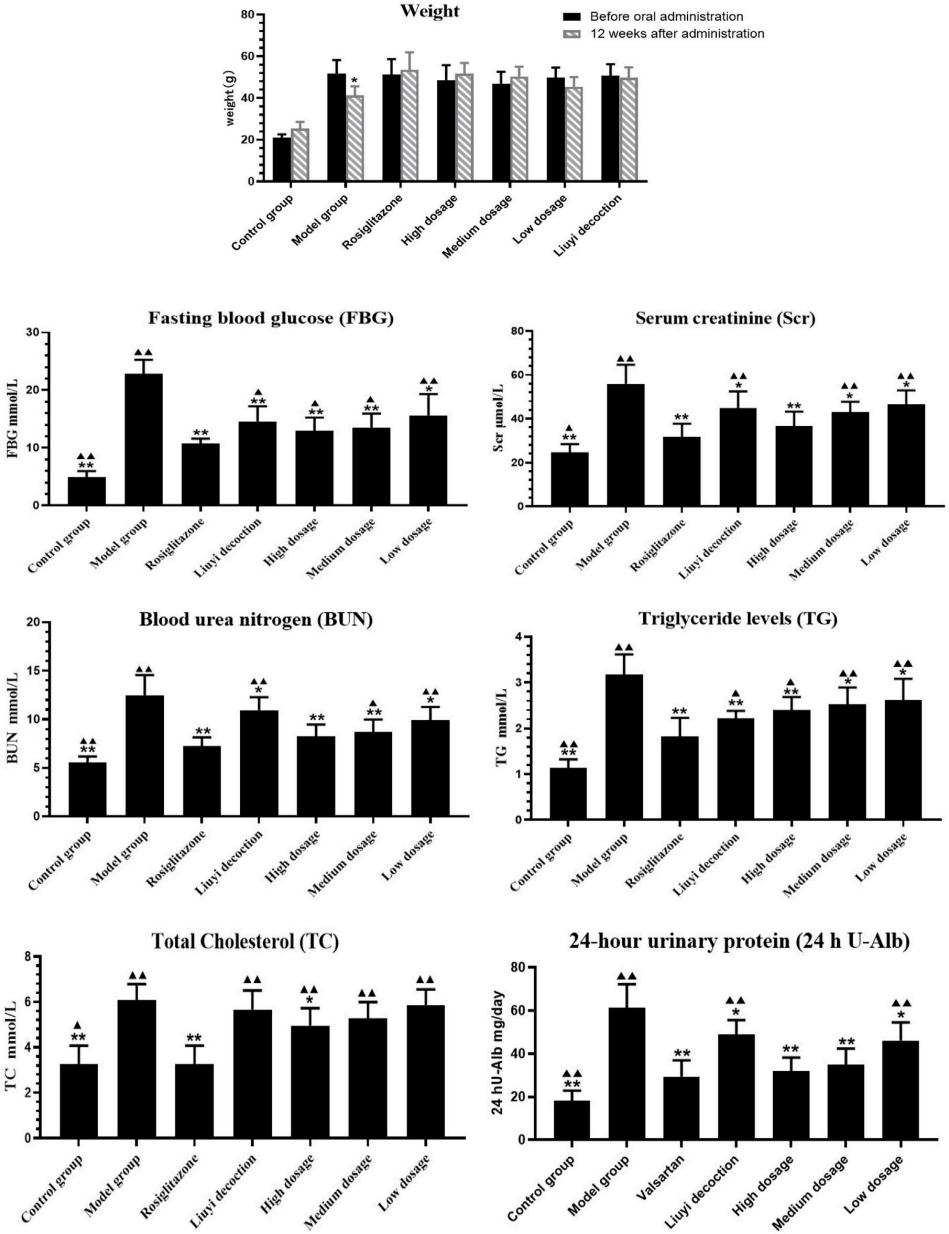
Results of biochemical indexes in each group (‾x±SD, *n* = 6). ps: vs. Model group, *P<0.05, **P<0.01; VS positive control group, ^▲^ *P*<0.05, ^▲▲^ *P*<0.01.

After 12 weeks, the weight of the model group was significantly decreased, the hair had lost its normal luster and was sparse, and the animals appeared less active. Compared with before administration, the weight of the HQD administration group was not significantly decreased, their fur remained shiny, and the animals were active (Figure 2).

Compared to those of the control group, 12-week-old diabetic mice showed increased FBG, BUN, Scr, TG, TC, and 24 h U-Alb levels (*p <* 0.05) (Figure 2). Diabetic mice treated with HQD showed lower biochemical parameters compared to untreated (*p <* 0.05). There was no significant difference in parameters between HQD and Huangqi Liuyi decoction treated animals (*p* > 0.05).

**FIGURE 3:**
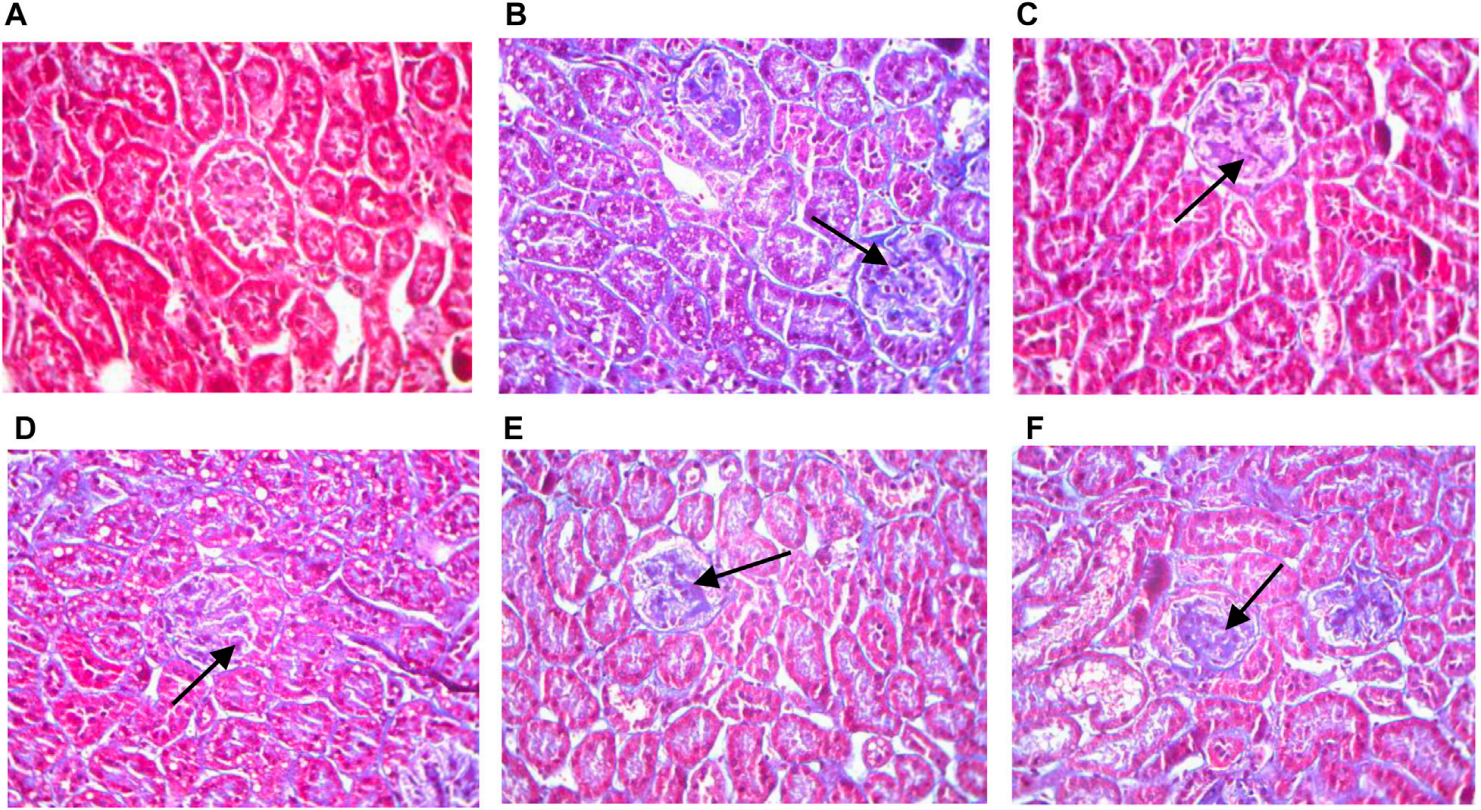
Kidneys pathological sectioning of various groups (Masson ×400). Control group **(A)**; model group **(B)**; high dosage **(C)**; medium dosage **(D)**; low dosage **(E)**; Huangqi Liuyi decoction **(F)**.

Masson’s trichrome stained collagen fibers blue, while muscle fibers, cytoplasm, cellulose, and keratin appeared red. Kidney sections from of 12-week-old db/db diabetic mice showed increased collagen fibers in the glomerular basement membrane (Figure 3). Animals treated with the HQD showed less kidney collagen compared to the model group.

**FIGURE 4:**
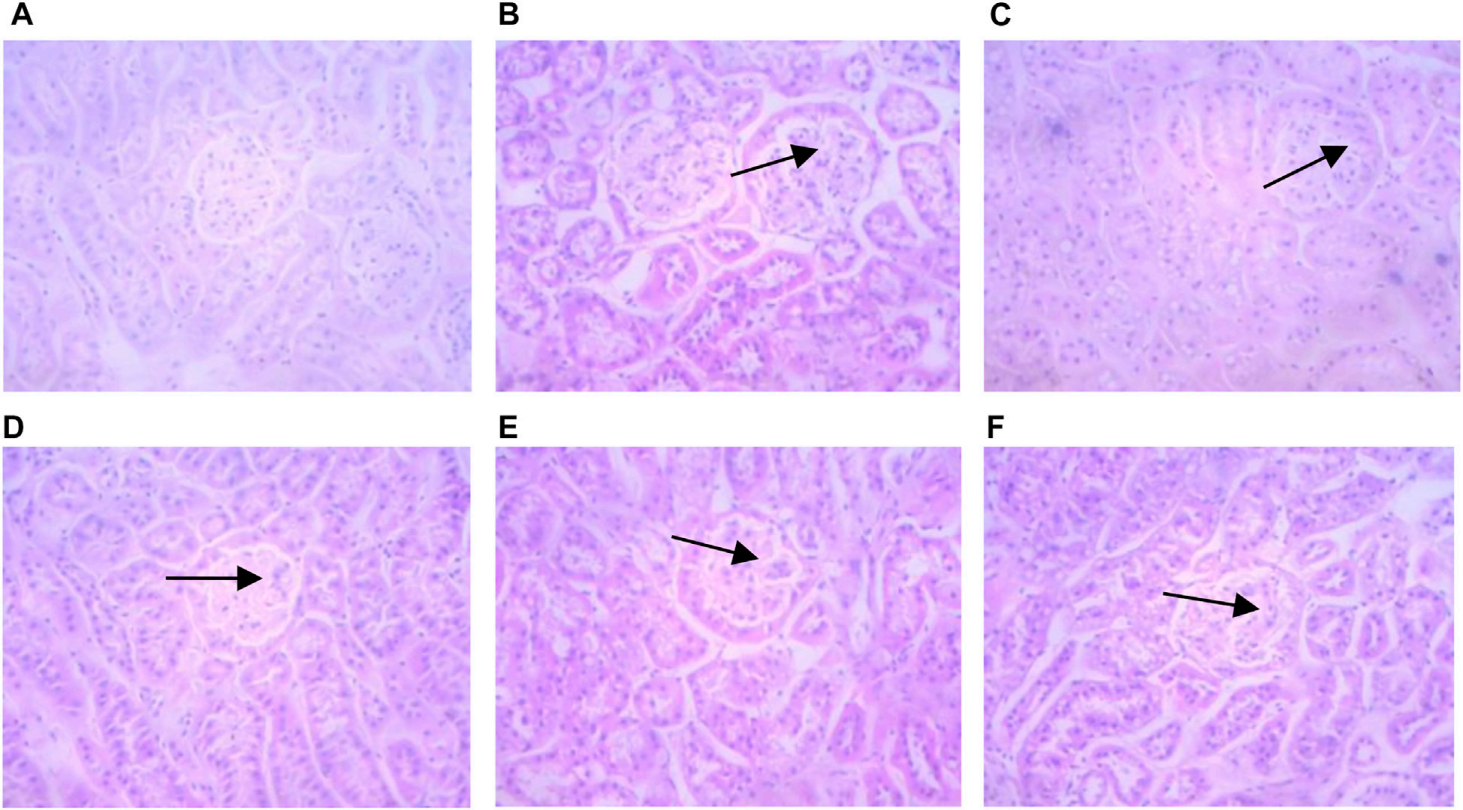
Kidneys pathological sectioning of various groups (HE ×400). Control group **(A)**; model group **(B)**; high dosage **(C)**; medium dosage **(D)**; low dosage **(E)**; Huangqi Liuyi decoction **(F)**.

In non-diabetic control mice, the glomerular structure showed no pathologic changes in the glomerular mesangial area (Figure 4). Conversely, kidneys from diabetic mice showed mesangial matrix deposition and thickening and nodular sclerosis of the glomerular capillary basement membrane. Diabetic mice treated with HQD showed less kidney damage compared to untreated.

### 3.2 Protein Expression of Collagen I, E-Cadherin, ɑ-Smooth Muscle Actin, and Vimentin

**FIGURE 5:**
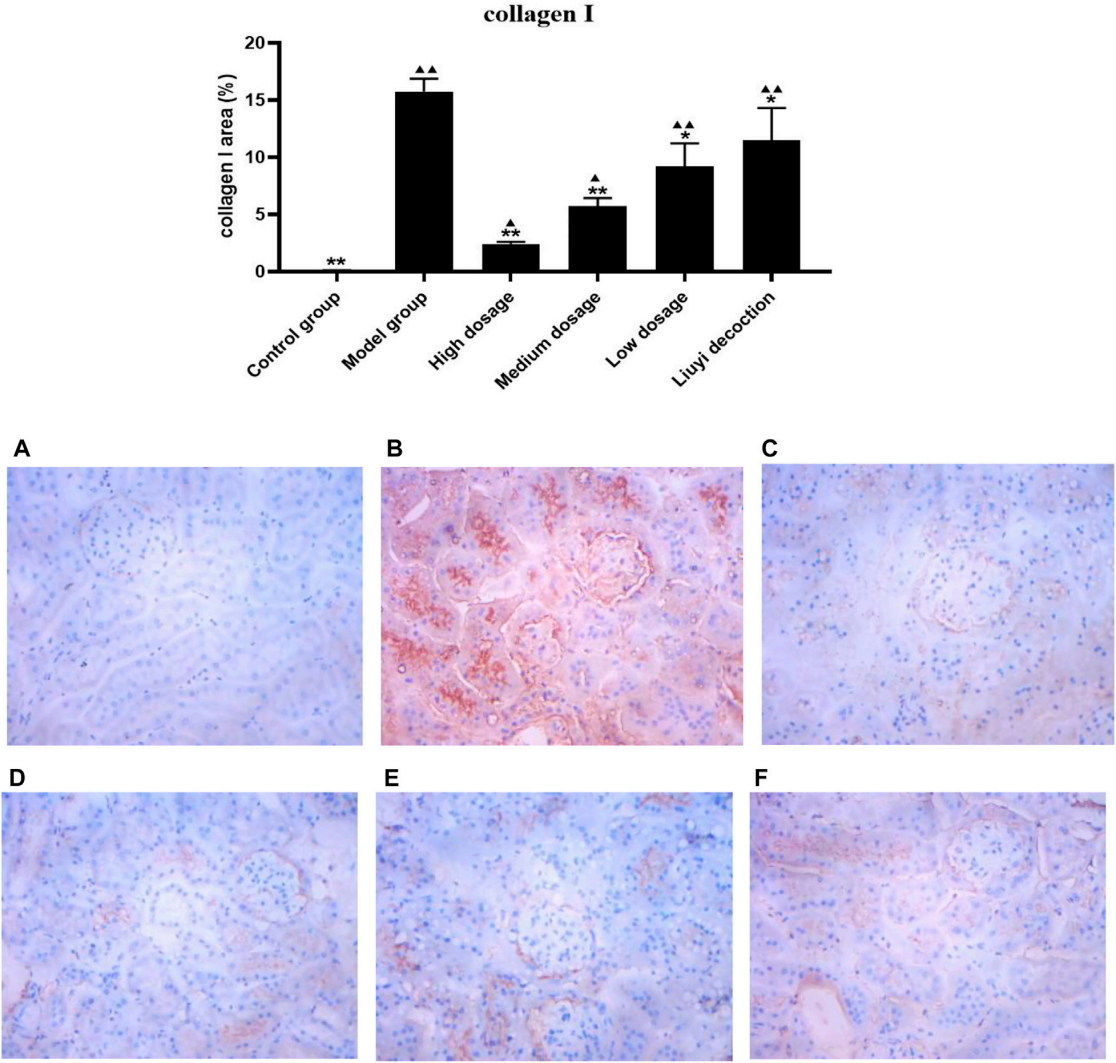
Effect of HQD on the expressions of collagen I in kidney (×400). Control group **(A)**; model group **(B)**; high dosage **(C)**; medium dosage **(D)**; low dosage **(E)**; Huangqi Liuyi decoction **(F)**. ps: vs. Model group, *P<0.05, **P<0.01; vs. control group, ^▲^ *P*<0.05, ^▲▲^ *P*<0.01 (x±SD, *n*=6).

**FIGURE 6:**
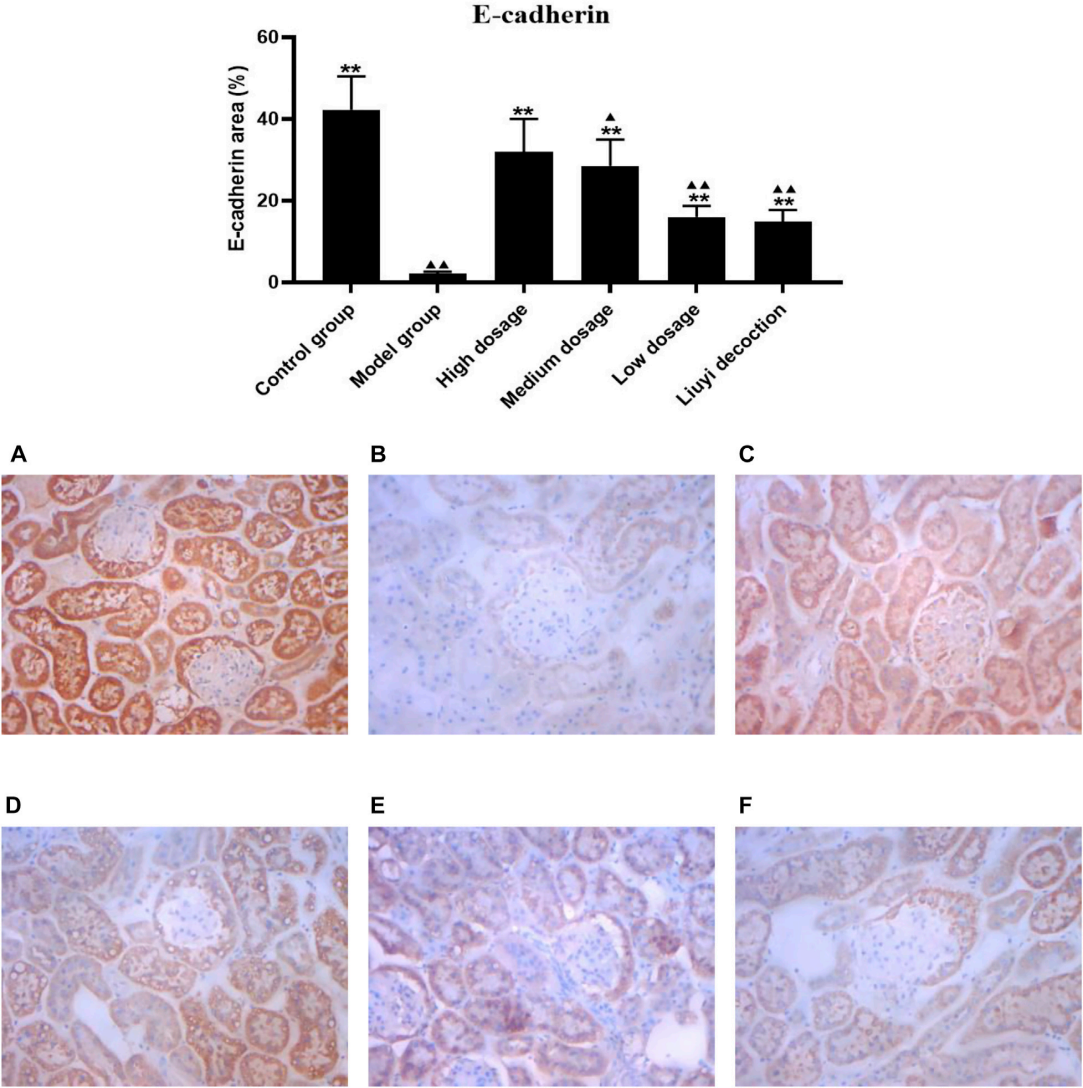
Effect of HQD on the expressions of E-cadherin in kidney (×400). Control group **(A)**; model group **(B)**; high dosage **(C)**; medium dosage **(D)**; low dosage **(E)**; Huangqi Liuyi decoction **(F)**. ps: vs. Model group, *P<0.05, **P<0.01; vs. control group, ^▲^ *P*<0.05, ^▲▲^ *P*<0.01 (x±SD, *n*=6).

**FIGURE 7:**
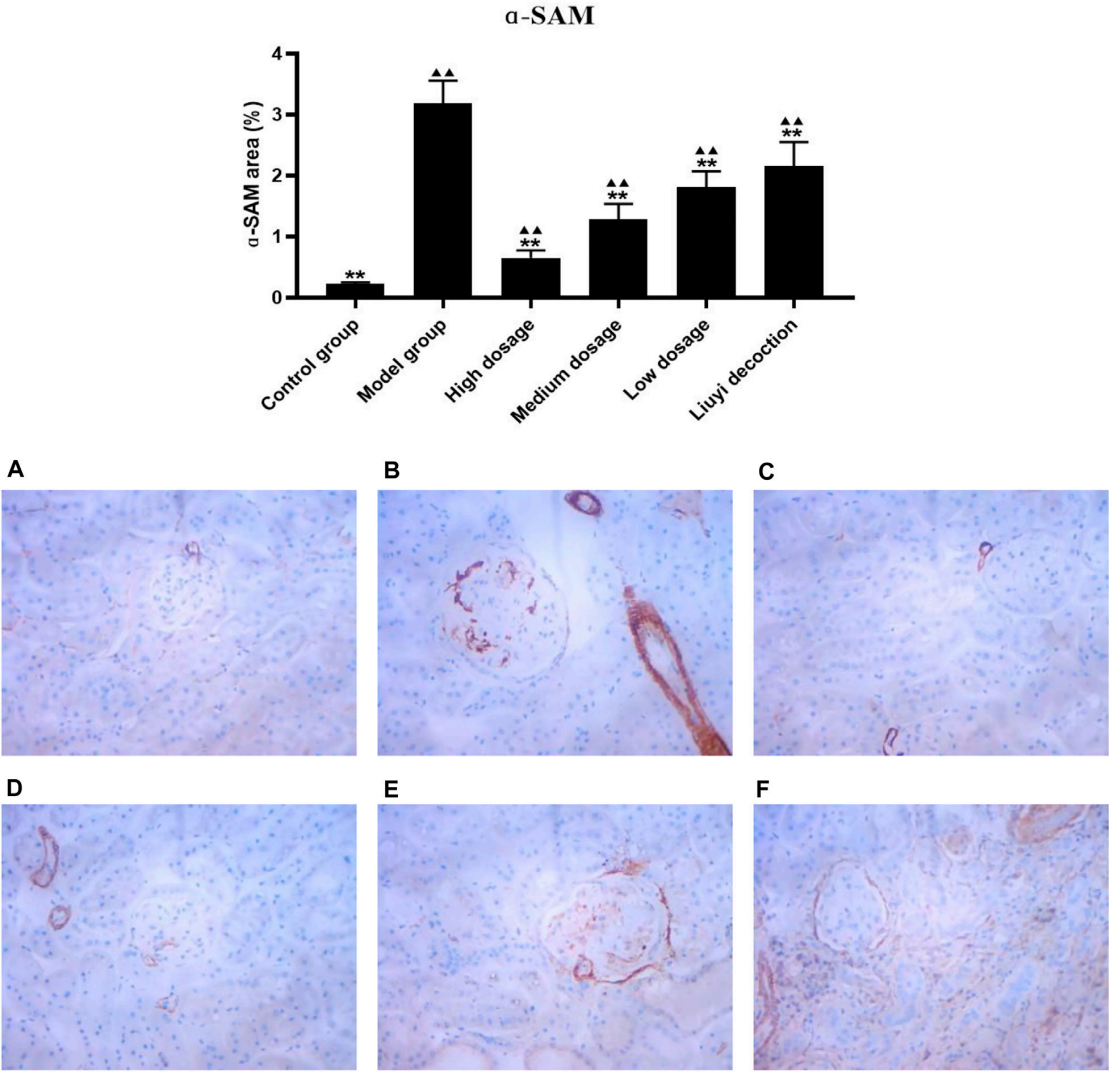
Effect of HQD on the expressions of ɑ-SAM in kidney (×400). Control group **(A)**; model group **(B)**; high dosage **(C)**; medium dosage **(D)**; low dosage **(E)**; Huangqi Liuyi decoction **(F)**. ps: vs. Model group, *P<0.05, **P<0.01; vs. control group, ^▲^ *P*<0.05, ^▲▲^ *P*<0.01 (x±SD, *n*=6).

**FIGURE 8:**
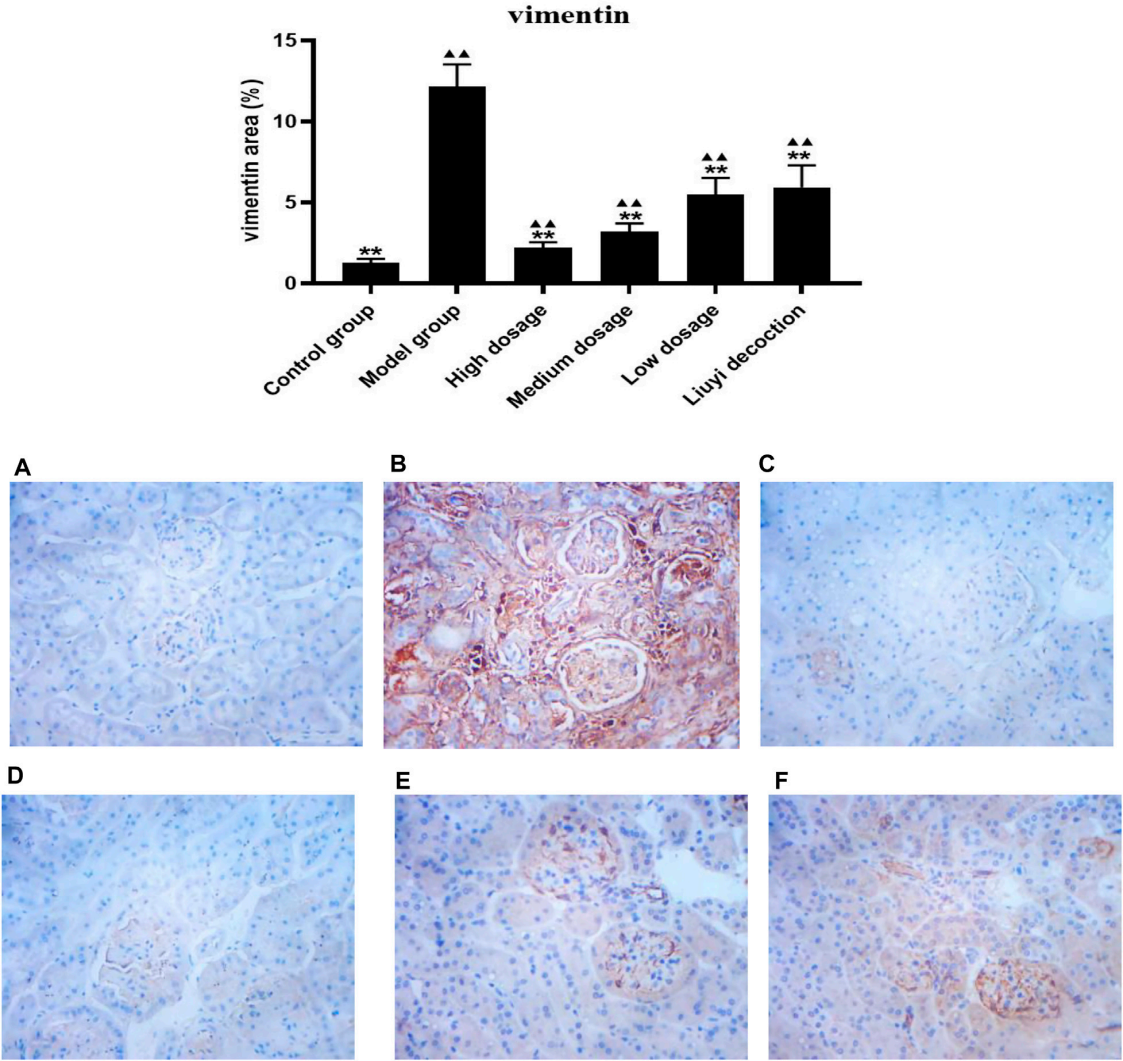
Effect of HQD on the expressions of vimentin in kidney (×400). Control group **(A)**; model group **(B)**; high dosage **(C)**; medium dosage **(D)**; low dosage **(E)**; Huangqi Liuyi decoction **(F)**. ps: vs. Model group, *P<0.05, **P<0.01; vs control group, ^▲^ *P*<0.05, ^▲▲^ *P*<0.01 (x±SD, *n*=6).

The semi-quantitative analysis of collagen I, E-cadherin, ɑ-SMA, and vimentin is showed in Figures 5–8. Expression of collagen I, ɑ-SMA, and vimentin was significantly increased (*p <* 0.01) and that of E-cadherin was significantly decreased (*p <* 0.01) in kidney sections from diabetic mice compared to kidney samples from non-diabetic mice. In contrast, diabetic animals treated with HDQ or decoction showed less kidney collagen I, ɑ-SMA, and vimentin (*p <* 0.05); and more E-cadherin (*p <* 0.01) versus samples from untreated diabetic mice.

### 3.3 Tissue Distribution of the Active Constituents of Huangqi Liuyi Decoction in Control and Diabetic Nephropathy Mice

#### 3.3.1 Method Validation of HPLC-MS/MS

##### 3.3.1.1 Specificity

**FIGURE 9:**
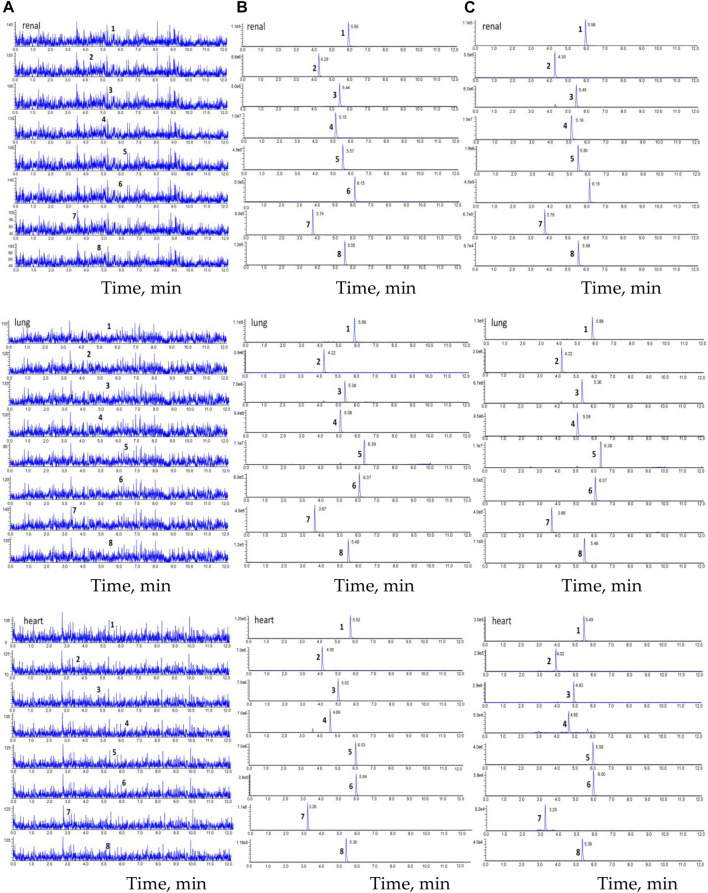
The chromatogram of HPLC-MS/MS. **(A)** blank tissue homogenate sample; **(B)** blank tissue homogenate sample spiked with six compounds and IS; **(C)** The tissue homogenate sample obtained at 30 min after oral HQD. (1. astragaloside IV, 2. calycosin-7-O-β-D-glucoside, 3. calycosin-glucuronide, 4. ononin, 5. formononetin, 6. glycyrrhetinic acid, 7. puerarin, and 8. digoxin).

Chromatograms of the blank tissue homogenate (e.g., heart, lung, and renal); blank tissue homogenate spiked with astragaloside IV, calycosin-7-O-β-D-glucoside, calycosin-glucuronide, ononin, formononetin, glycyrrhizic acid, and IS; and tissue homogenate obtained after oral administration of HQD are displayed in Figure 9. Good separation was observed among the ingredients, and there was no interference from the endogenous substances in the determination of the six ingredients and IS.

##### 3.3.1.2 Calibration Curves and Linearity

The typical equation of linearity ranges and calibration curves for the six ingredients are shown in Table 2 (e.g., heart, lungs, and kidneys). The lowest concentration in the calibration curve was defined as the lower limit of quantification. The results show that all correlation coefficients are higher than 0.999, indicating that the concentrations of the six ingredients in mouse tissues correlated well within the linearity ranges.

**TABLE 2:** Calibration curve and LOQ of six ingredients in tissue homogenate samples.

Biological sample	Analyte	Calibration curve	r	Linear range	LLOQ ng/g	LOD ng/g
Kidney	Astragaloside IV	y = 0.4594x ± 1.0711	0.9990	5.34–1335	5.34	0.1
	Calycosin-7-O-β-D-glucoside	y = 0.1446x ± 0.1856	0.9992	0.25–50.5	0.25	0.04
	Calycosin-glucuronide	y = 0.1912x ± 0.0975	0.9991	2.60–519	2.60	0.06
	Formononetin	y = 0.0851x ± 0.2989	0.9992	1.05–104.6	1.05	0.02
	Ononin	y = 0.0968x ± 0.1290	0.9991	1.01–101.4	1.01	0.15
	Glycyrrhizic acid	y = 0.0974x ± 0.1794	0.9994	1.02–1018	1.02	0.34
Heart	Astragaloside IV	y = 0.1207x ± 0.2282	0.9991	5.34–1335	5.34	0.1
	Calycosin-7-O-β-D-glucoside	y = 0.0586x ± 0.1121	0.9992	0.25–50.5	0.25	0.04
	Calycosin-glucuronide	y = 0.5242x ± 0.2481	0.9993	2.60–519	2.60	0.06
	Formononetin	y = 0.1101x ± 0.1341	0.9990	1.05–104.6	1.05	0.02
	Ononin	y = 0.0899x ± 0.1431	0.9992	1.01–101.4	1.01	0.15
	Glycyrrhizic acid	y = 0.1445x ± 0.0502	0.9991	1.02–1018	1.02	0.34
	Astragaloside IV	y = 0.6027x ± 0.8407	0.9992	5.34–1335	5.34	0.1
	Calycosin-7-O-β-D-glucoside	y = 0.2430x ± 0.3439	0.9991	0.25–50.5	0.25	0.04
Lung	Calycosin-glucuronide	y = 0.0736x ± 0.0819	0.9991	2.60–519	2.60	0.06
	Formononetin	y = 0.1031x ± 0.3993	0.9990	1.05–104.6	1.05	0.02
	Ononin	y = 0.0855x ± 0.0551	0.9992	1.01–101.4	1.01	0.15
	Glycyrrhizic acid	y = 0.1740x ± 0.1234	0.9993	1.02–1018	1.02	0.34

##### 3.3.1.3 Accuracy and Precision

Intraday and interday precision and accuracy (e.g., heart, lungs, and kidneys) are summarized in Table 3. The intraday and interday RSDs were below 20.0%. Thus, the accuracy and precision results met the requirements of biological sample detection.

**TABLE 3:** Precision and accuracy of ingredients in tissue homogenate samples (‾x ± SD; *n* = 6).

Analyte	Biological sample	Concentration of analyte (ng/g)	Mean ± SD (ng/g)	Accuracy (%)	Interday precision RSD (%)	Intraday precision RSD (%)
Astragaloside IV	Heart	26.7	29.01 ± 3.22	108.6 ± 3.83	11.11	6.82
		133.5	142.1 ± 12.68	106.5 ± 9.41	8.92	10.75
		267	264.7 ± 41.44	99.12 ± 15.62	15.66	17.63
	Lung	26.7	25.09 ± 3.27	106.8 ± 13.04	13.04	11.81
		133.5	122.9 ± 14.03	105.2 ± 11.56	11.41	10.50
		267	259.1 ± 19.35	97.10 ± 3.80	7.47	5.77
	Kidney	26.7	27.8 ± 4.45	96.44 ± 16.28	16.00	15.38
		133.5	136.6 ± 6.32	107.7 ± 11.21	4.63	11.97
		267	276.8 ± 22.67	95.61 ± 10.75	8.19	6.90
Calycosin-7-O-β-D-glucoside	Heart	2.53	2.53 ± 0.41	100.05 ± 9.76	16.15	7.53
		12.63	10.92 ± 1.29	86.44 ± 5.42	11.81	12.36
		25.25	24.45 ± 3.97	96.83 ± 15.82	16.23	14.14
	Lung	2.53	2.56 ± 0.18	104.3 ± 8.18	7.04	10.53
		12.63	13.54 ± 1.25	96.60 ± 16.84	9.20	9.66
		25.25	22.93 ± 2.25	97.59 ± 7.69	9.79	7.89
	Kidney	2.53	2.48 ± 0.21	98.50 ± 6.85	8.53	6.95
		12.63	13.82 ± 0.51	106.3 ± 13.87	3.67	14.93
		25.25	22.94 ± 0.32	97.23 ± 5.23	1.38	5.11
Calycosin-glucuronide	Heart	5.19	5.88 ± 0.65	113.3 ± 4.60	11.07	11.87
		51.9	46.24 ± 8.70	87.16 ± 4.78	18.79	11.26
		259.5	262.9 ± 49.63	101.2 ± 9.84	18.87	9.73
	Lung	5.19	4.77 ± 0.65	102.6 ± 7.80	13.60	11.30
		51.9	58.32 ± 4.32	101.7 ± 8.60	7.40	17.58
		259.5	272.1 ± 10.36	99.67 ± 16.15	3.81	16.20
	Kidney	5.19	5.03 ± 0.69	101.3 ± 10.01	13.81	16.78
		51.9	47.84 ± 7.72	104.8 ± 6.42	16.14	5.72
		259.5	259.1 ± 22.77	108.8 ± 10.65	8.79	11.05
Ononin	Heart	5.07	5.79 ± 0.59	114.24 ± 4.48	10.18	11.71
		25.35	27.19 ± 5.07	109.1 ± 7.24	18.62	12.18
		50.7	46.59 ± 6.07	91.90 ± 7.03	13.03	9.70
	Lung	5.07	5.29 ± 0.27	89.60 ± 6.50	5.02	3.91
		25.35	21.27 ± 0.95	116.3 ± 1.28	4.45	1.19
		50.7	45.35 ± 3.69	98.95 ± 7.56	8.13	6.84
	Kidney	5.07	4.35 ± 0.15	88.58 ± 4.94	3.48	7.70
		25.35	22.59 ± 2.5	108.6 ± 5.53	11.09	6.41
		50.7	48.85 ± 1.88	109.2 ± 3.61	3.85	1.60
Formononetin	Heart	5.23	4.84 ± 0.29	92.54 ± 4.85	6.10	9.74
		26.15	24.95 ± 3.45	95.40 ± 13.35	13.83	18.29
		52.3	56.17 ± 8.9	107.4 ± 11.02	15.84	12.63
	Lung	5.23	4.61 ± 0.21	101.9 ± 9.37	4.56	16.32
		26.15	23.38 ± 1.79	105.1 ± 6.40	7.66	15.76
		52.3	54.25 ± 6.31	110.1 ± 4.96	11.64	7.40
	Kidney	5.23	4.52 ± 0.57	112.4 ± 5.94	12.56	11.49
		26.15	22.37 ± 2.99	105.3 ± 11.75	13.36	5.24
		52.3	47.26 ± 5.98	113.7 ± 4.49	12.65	6.48
Glycyrrhizic acid	Heart	50.9	48.39 ± 4.82	95.06 ± 4.49	9.97	9.41
		254.5	285.3 ± 33.65	112.1 ± 5.22	11.79	6.44
		509	525.1 ± 55.03	103.1 ± 10.81	10.48	18.69
	Lung	50.9	55.39 ± 3.4	102.3 ± 8.13	6.13	2.15
		254.5	246.4 ± 22.15	109.3 ± 9.61	8.99	4.49
		509	526.9 ± 86.3	99.56 ± 11.70	16.38	16.25
	Kidney	50.9	53.85 ± 2.94	99.35 ± 7.67	5.46	6.08
		254.5	256.1 ± 10.78	107.4 ± 11.69	4.21	5.76
		509	540.9 ± 38.62	106.2 ± 7.29	7.14	15.89

##### 3.3.1.4 Extraction Efficiency and Matrix Effect

Results of the extraction efficiency and matrix effect analyses are shown in Table 4 (e.g., heart, lungs, and kidneys). As noted, the recoveries of the six ingredients were precise, consistent, and reproducible at different concentration levels in various tissue samples, and there was no significant matrix interference.

**TABLE 4:** The mean recoveries of ingredients in tissue homogenate samples (x ± SD; *n* = 6).

Analyte	Concentration of analyte (ng/ml)	Biological sample	Extraction recovery (%)	RSD%	Matrix effect (%)	RSD%
Astragaloside IV	133.5	Heart	99.59 ± 16.18	16.25	86.82 ± 5.15	5.14
		Lung	112.4 ± 9.66	8.60	97.50 ± 8.32	8.53
		Kidney	84.96 ± 3.76	4.43	106.9 ± 6.48	6.06
Calycosin-7-O-β-D-glucoside	12.63	Heart	95.27 ± 13.23	13.89	108.86 ± 10.8	12.68
		Lung	95.24 ± 7.04	7.39	116.9 ± 2.06	1.76
		Kidney	92.44 ± 5.99	6.48	115.2 ± 2.39	2.08
Calycosin-glucuronide	51.9	Heart	107.3 ± 9.56	8.90	99.45 ± 16.55	16.63
		Lung	89.07 ± 8.11	9.12	108.4 ± 9.14	8.43
		Kidney	90.30 ± 7.14	7.89	99.57 ± 6.12	6.15
Formononetin	26.15	Heart	114.3 ± 3.91	3.42	113.6 ± 5.31	4.67
		Lung	108.0 ± 8.63	7.98	107.0 ± 4.82	4.51
		Kidney	115.7 ± 4.64	4.02	98.60 ± 15.31	15.53
Ononin	25.35	Heart	112.7 ± 5.33	4.73	83.12 ± 2.86	3.44
		Lung	97.71 ± 2.49	2.55	93.04 ± 12.25	13.16
		Kidney	98.44 ± 2.39	2.43	86.87 ± 3.29	3.78
Glycyrrhizic acid	254.5	Heart	83.60 ± 2.25	2.69	102.7 ± 15.92	15.51
		Lung	89.35 ± 2.08	2.33	102.8 ± 5.88	5.73
		Kidney	96.60 ± 3.64	3.77	97.65 ± 4.60	4.71

##### 3.3.1.5 Stability

Results of the stability analysis are shown in Table 5 (e.g., heart, lungs, and kidneys). The stability test results indicated that the mouse tissue samples showed good stability under the three different conditions with a 10% concentration variation compared to the initial values.

**TABLE 5:** Stability of ingredients in tissue homogenate samples (x ± SD; *n* = 6).

Analyte	Biological sample	Concentration of analyte (ng/g)	Sampler 4 h		−20°C 48 h		Three freeze-thaw	
			Mean ± SD (ng/ml)	RSD%	Mean ± SD (ng/ml)	RSD%	Mean ± SD (ng/ml)	RSD%
Astragaloside IV	Heart	26.7	28.02 ± 2.08	7.44	27.57 ± 2.51	9.12	26.63 ± 3.83	14.39
		133.5	141.6 ± 5.76	4.07	121.0 ± 12.09	9.99	141.8 ± 15.94	11.24
		267	276.3 ± 27.25	9.86	248.4 ± 42.35	17.05	263.3 ± 40.2	15.27
	Lung	26.7	27.06 ± 1.74	6.43	27.7 ± 4.8	17.32	26.76 ± 2.54	9.50
		133.5	136.7 ± 14.6	10.68	134.7 ± 23.05	17.11	136.8 ± 20.39	14.90
		267	258.4 ± 22.45	8.69	257.0 ± 9.82	3.82	280.7 ± 17.52	6.24
	Kidney	26.7	27.35 ± 1.46	5.34	27.83 ± 5.13	18.44	27.17 ± 1.81	6.65
		133.5	150.1 ± 16.42	10.94	139.9 ± 8.52	6.09	137.8 ± 14.78	10.73
		267	283.0 ± 21.23	7.5	260.0 ± 20.38	7.84	253.7 ± 27.29	10.76
Calycosin-7-O-β-D-glucoside	Heart	2.53	2.29 ± 0.45	19.88	2.39 ± 0.22	9.33	2.67 ± 0.44	16.47
		12.63	12.15 ± 0.95	7.8	12.43 ± 0.89	7.16	12.67 ± 0.9	7.07
		25.25	26.25 ± 1.19	4.53	26.54 ± 2.66	10.01	23.84 ± 2.25	9.43
	Lung	2.53	2.6 ± 0.1	3.68	2.71 ± 0.12	4.45	2.52 ± 0.23	9.01
		12.63	13.29 ± 1.09	8.20	12.35 ± 0.6	4.85	12.23 ± 1.26	10.29
		25.25	25.58 ± 1.85	7.24	24.85 ± 1.64	6.58	24.42 ± 2.3	9.41
	Kidney	2.53	2.75 ± 0.24	8.87	2.39 ± 0.23	9.72	2.37 ± 0.37	15.73
		12.63	12.76 ± 1.21	9.52	13.18 ± 0.78	5.89	12.47 ± 0.73	5.88
		25.25	24.7 ± 1.22	4.94	24.32 ± 1.11	4.55	24.15 ± 1.16	4.81
Calycosin-glucuronide	Heart	5.19	4.9 ± 0.71	14.33	4.72 ± 0.67	14.21	4.98 ± 0.38	7.59
		51.9	53.51 ± 3.09	5.78	50.5 ± 3.89	7.70	52.31 ± 3.24	6.19
		259.5	273.1 ± 21.63	7.92	248.1 ± 16.15	6.51	246.4 ± 21.17	8.59
	Lung	5.19	5.14 ± 0.79	15.27	4.86 ± 1.15	23.54	4.77 ± 0.89	18.68
		51.9	53.94 ± 2.69	4.98	51.6 ± 7.51	14.55	50.42 ± 7.7	15.28
		259.5	254.4 ± 22.24	8.74	249.9 ± 22.29	8.92	253.7 ± 40.95	16.14
	Kidney	5.19	4.87 ± 0.88	18.11	5.03 ± 0.59	11.65	4.77 ± 0.46	9.69
		51.9	51.3 ± 1.58	3.08	51.28 ± 5.33	10.40	53.06 ± 5.75	10.84
		259.5	241.6 ± 29.12	12.05	247.6 ± 23.25	9.39	250.3 ± 29.34	11.72
Ononin	Heart	5.07	5.05 ± 0.88	17.48	5.10 ± 0.59	11.54	5.54 ± 0.31	5.58
		25.35	23.75 ± 3.76	15.81	24.05 ± 4.17	17.35	24.17 ± 3.67	15.17
		50.7	49.37 ± 2.58	5.22	50.61 ± 4.73	9.35	49.36 ± 2.62	5.31
	Lung	5.07	5.32 ± 0.49	9.29	5.1 ± 0.19	3.68	5.03 ± 0.42	8.26
		25.35	24.52 ± 0.27	1.12	23.22 ± 4.98	21.45	24.86 ± 1.1	4.44
		50.7	49.15 ± 2.36	4.81	50.88 ± 2.08	4.08	48.89 ± 2.51	5.14
	Kidney	5.07	5.19 ± 0.38	7.23	5.56 ± 0.36	6.48	4.95 ± 0.62	12.54
		25.35	25.98 ± 2.88	11.08	23.37 ± 1.5	6.40	23.25 ± 1.03	4.43
		50.7	49.97 ± 3.04	6.09	51.9 ± 3.07	5.92	49.96 ± 3.16	6.32
Formononetin	Heart	5.23	4.99 ± 0.95	19.03	4.95 ± 0.66	13.31	5.32 ± 0.92	17.31
		26.15	28.08 ± 3.88	13.83	25.2 ± 3.60	14.29	24.6 ± 2.94	11.94
		52.3	47.88 ± 5.64	11.77	48.03 ± 3.61	7.52	49.59 ± 2.52	5.09
	Lung	5.23	5.56 ± 0.73	13.13	5.03 ± 0.88	17.48	4.8 ± 0.82	17.09
		26.15	28.13 ± 5.28	18.79	24.02 ± 4.72	19.67	26.46 ± 4.32	16.34
		52.3	54.71 ± 5.32	9.73	54.9 ± 4.66	8.48	48.82 ± 8.6	17.62
	Kidney	5.23	5.54 ± 0.74	13.32	5.06 ± 1.03	20.35	5.12 ± 0.65	12.63
		26.15	26.81 ± 2.04	7.62	24.24 ± 2.42	10.00	24.55 ± 2.84	11.55
		52.3	54.25 ± 7.07	13.04	48.03 ± 5.16	10.75	49.98 ± 9.62	19.24
Glycyrrhizic acid	Heart	50.9	51.38 ± 0.82	1.60	54.32 ± 6.22	11.45	53.92 ± 4.38	8.13
		254.5	276.9 ± 41.22	14.88	272.1 ± 37.85	13.91	267.5 ± 16.27	6.08
		509	514.5 ± 34.16	6.64	516.6 ± 38.02	7.36	510.5 ± 38.03	7.45
	Lung	50.9	47.41 ± 6.34	13.37	47.21 ± 3.6	7.63	48.9 ± 3.34	6.83
		254.5	232.1 ± 16.92	7.29	236.4 ± 12.72	5.38	257.8 ± 23.04	8.94
		509	502.3 ± 18.54	3.69	504.5 ± 23.51	4.66	501.9 ± 39.3	7.83
	Kidney	50.9	49.16 ± 1.79	3.65	48.28 ± 3.7	7.67	49.34 ± 2.38	4.82
		254.5	243.8 ± 13.46	5.52	234.8 ± 10.26	4.37	239.2 ± 11.75	4.91
		509	504.3 ± 28.95	5.74	507.7 ± 33.35	6.57	499.9 ± 37.44	7.49

#### 3.3.2 Tissue Distribution Study

**FIGURE 10:**
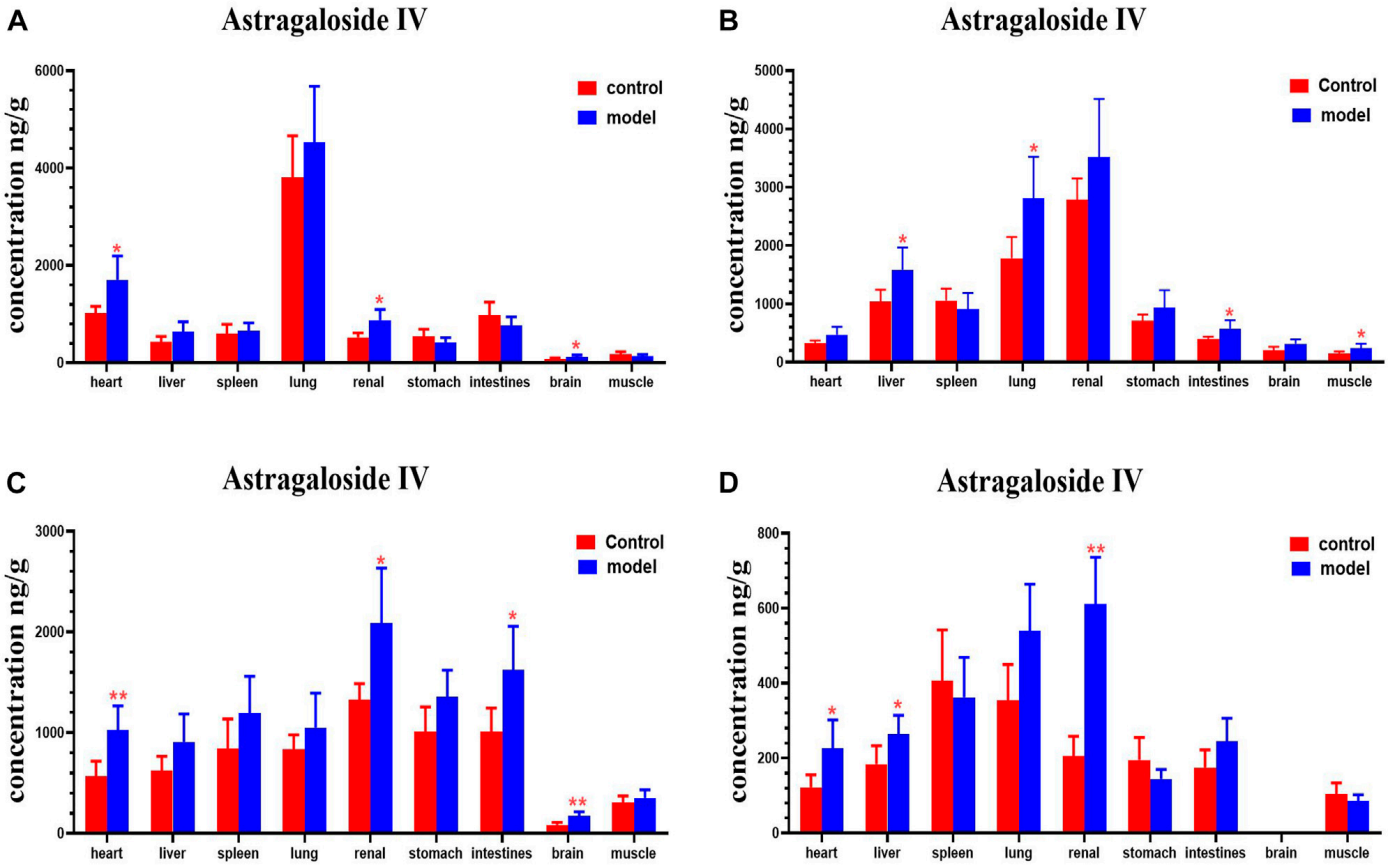
Content of astragaloside IV in different mouse tissue homogenate of the normal and DN models at four different time points after intragastric administration of HQD (x±SD, *n*=8), **(A)** 30 min, **(B)** 2 h, **(C)** 4 h, **(D)** 8 h. Ps: *P < 0.05, **P < 0.01 vs. normal group.

**FIGURE 11:**
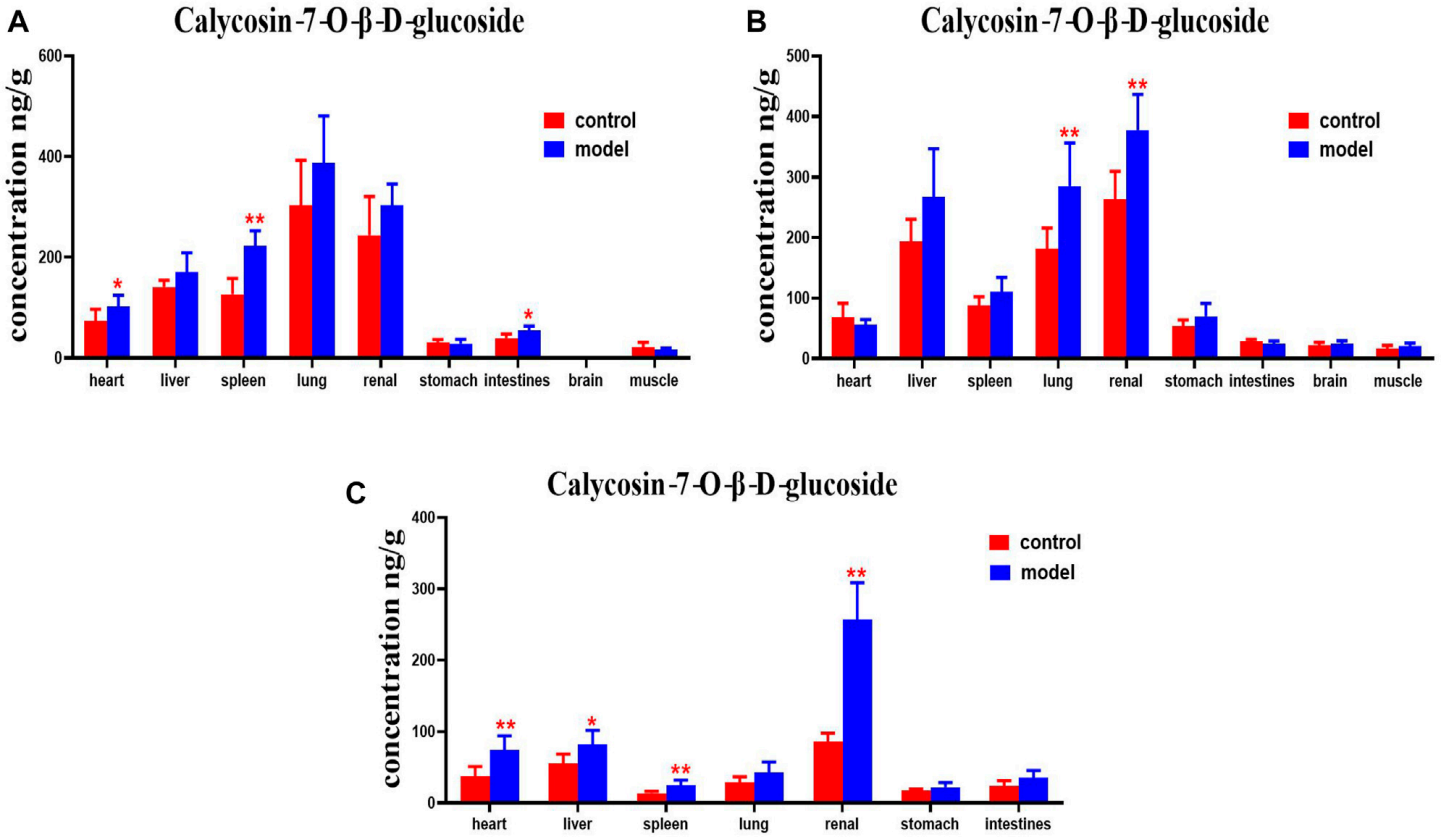
Content of calycosin-7-O-β-D-glucoside in different mouse tissue homogenate of the normal and DN models at four different time points after intragastric administration of HQD (x±SD, *n*=8), **(A)** 30 min, **(B)** 2 h, **(C)** 4 h. Ps: ^*^ *P* < 0.05, ^**^ *P* < 0.01 vs. normal group.

**FIGURE 12:**
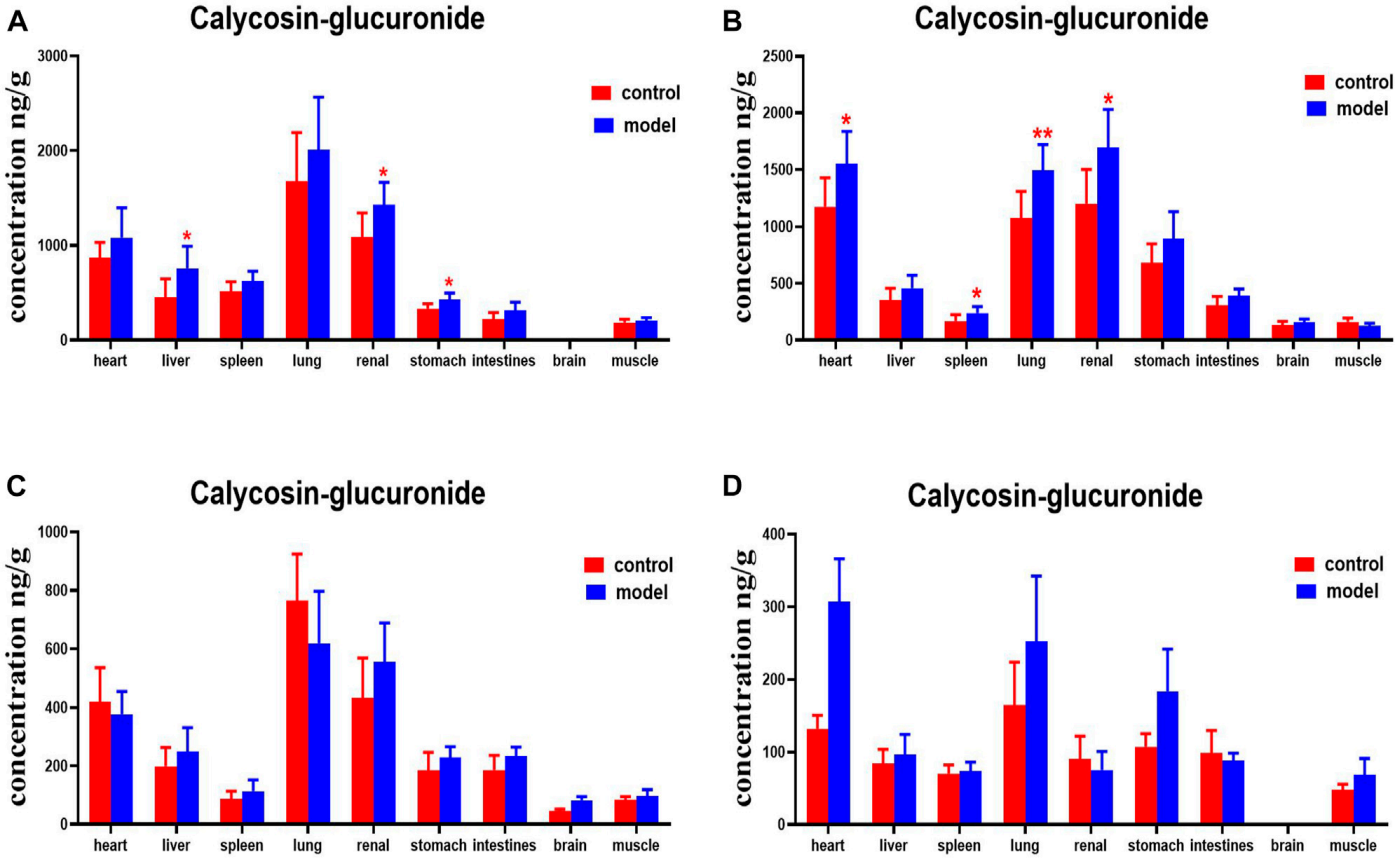
Content of calycosin-glucoside in different mouse tissue homogenate of the normal and DN models at four different time points after intragastric administration of HQD (x±SD, *n*=8), **(A)** 30 min, **(B)** 2 h, **(C)** 4 h, **(D)** 8 h. Ps: ^*^ *P* < 0.05, ^**^ *P* < 0.01 vs. normal group.

**FIGURE 13:**
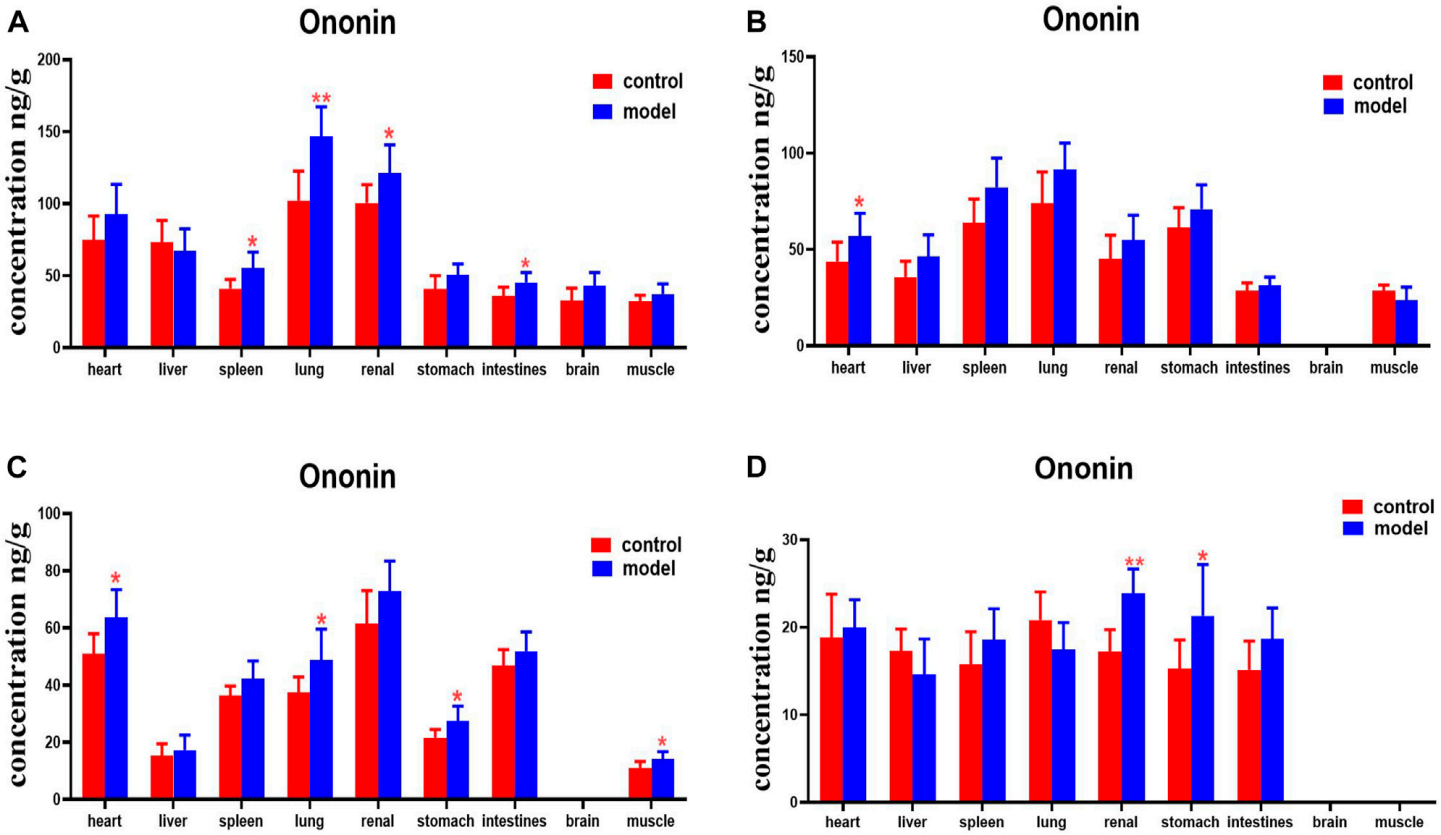
Content of ononin in different mouse tissue homogenate of the normal and DN models at four different time points after intragastric administration of HQD (x±SD, *n*=8), **(A)** 30 min, **(B)** 2 h, **(C)** 4 h, **(D)** 8 h. Ps: ^*^ *P* < 0.05, ^**^ *P* < 0.01 vs. normal group.

**FIGURE 14:**
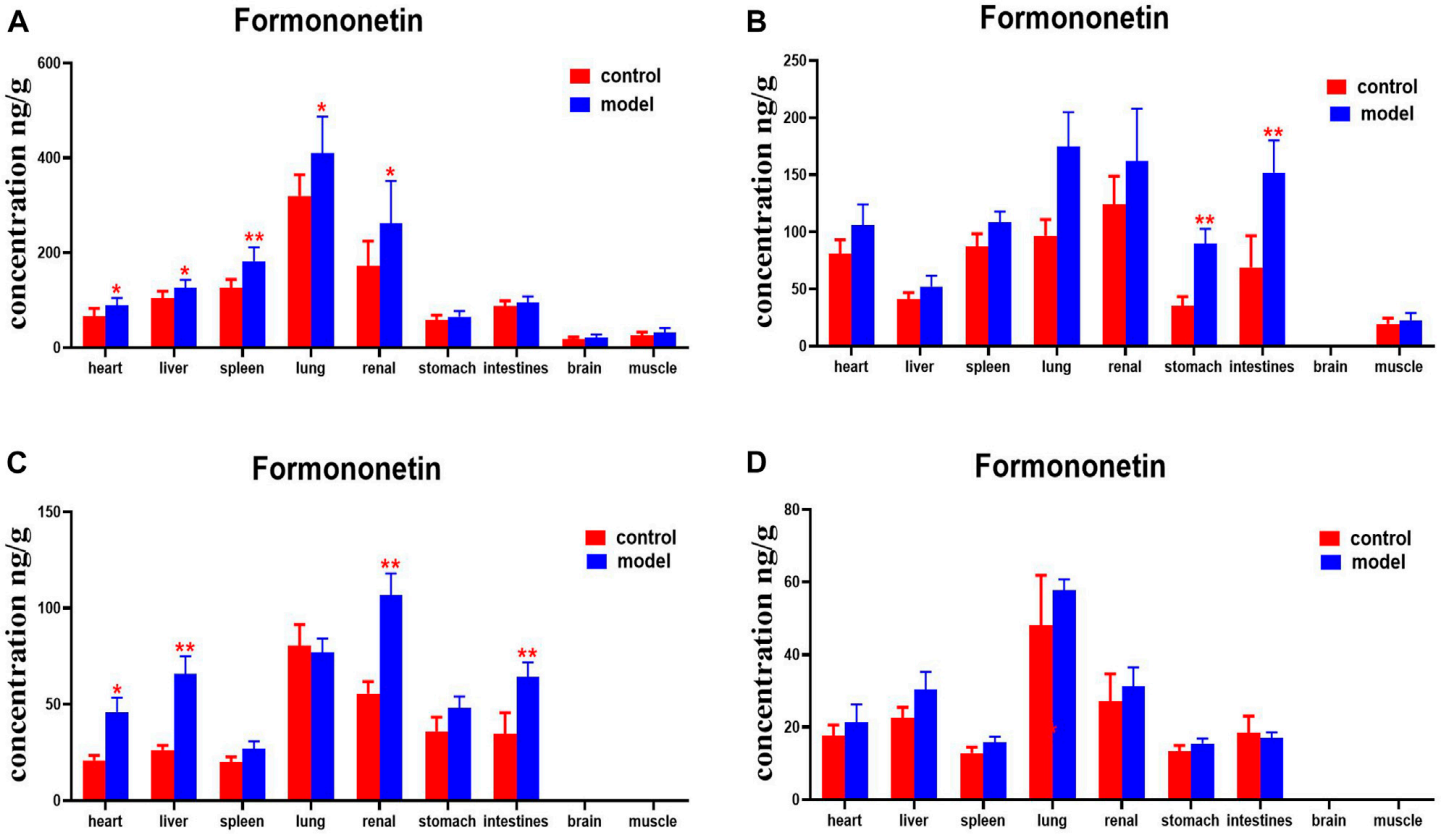
Content of formononetin in different mouse tissue homogenate of the normal and DN models at four different time points after intragastric administration of HQD (x±SD, *n*=8), **(A)** 30 min, **(B)** 2 h, **(C)** 4 h, **(D)** 8 h. Ps: ^*^ *P* < 0.05, ^**^ *P* < 0.01 vs. normal group.

**FIGURE 15:**
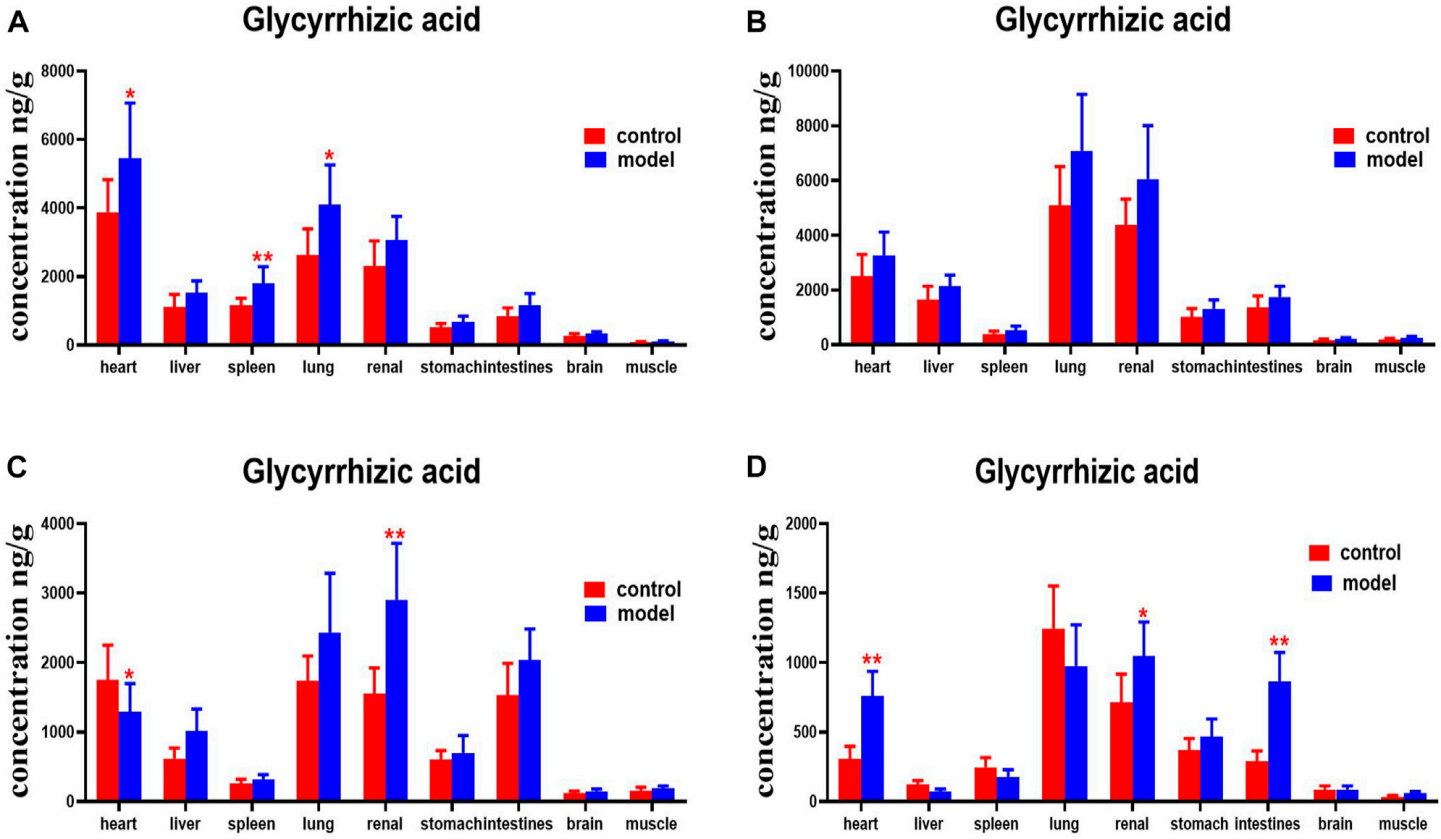
Content of glycyrrhetinic acid in different mouse tissue homogenate of the normal and DN models at four different time points after intragastric administration of HQD (x±SD, *n*=8), **(A)** 30 min, **(B)** 2 h, **(C)** 4 h, **(D)** 8 h. Ps: ^*^ *P* < 0.05, ^**^ *P* < 0.01 vs normal group.

Following a single oral administration of HQD, the distribution of the six ingredients (astragaloside IV, calycosin-7-O-β-D-glucoside, calycosin glucuronide, ononin, formononetin, and glycyrrhizic acid) between the normal and DN mouse groups were analyzed at different time points (30 min and 2, 4, and 8 h). The six ingredients were detected in all tissue samples from diabetic and non-diabetic mice (Figures 10–(15). Astragaloside IV, calycosin-7-O-β-D- glucoside, calycosin glucuronide, ononin, formononetin, and glycyrrhizic acid were found highest in the lungs and kidneys. However, the six active ingredients were minimally found in the brain. As expected, tissue distribution of all ingredients decreased with time.

Compared with the normal group, astragaloside IV levels in the hearts, kidney, and brains of mice with DN were higher at 30 min; levels of astragaloside IV in the livers, lungs, intestines, and muscles of mice with DN were higher at 2 h. Astragaloside IV levels in the hearts, kidneys, intestines and brains of mice with DN were higher at 4 h. Levels of astragaloside IV in the hearts, livers and kidneys of mice with DN were higher at 8 h (Figure 10).

Compared with the normal group, DN mice had higher levels of calycosin-7-O-β-D-glucoside in their hearts, spleens and intestines at 30 min. Calycosin-7-O-β-D-glucoside levels in the lungs and kidneys of DN mice were higher at 2 h. Levels of calycosin-7-O-β-D-glucoside in the hearts, livers, spleen, and kidneys of DN mice were higher at 4 h (Figure 11).

DN mice had higher levels of calycosin-glucoside in their livers, kidneys, and stomachs than the normal group at 30 min. Levels of calycosin-glucoside in the hearts, spleens, lungs, and kidneys of mice with DN were higher at 2 h (Figure 12).

Ononin levels were higher in the spleens, lungs, kidneys, and intestines of DN mice at 30 min, compared with the normal group. DN mice had higher levels of ononin in their hearts at 2 h. Levels of ononin in their hearts, lungs, stomachs, and muscles were higher at 4 h. Ononin levels in the kidneys and stomachs were higher at 8 h (Figure 13).

Compared with the normal group, DN mice had higher levels of formononetin in their hearts, livers, spleens, lungs, and kidneys at 30 min. Formononetin levels were also higher in their stomachs and intestines at 2 h. Levels of formononetin in the hearts, livers, kidneys and intestines of DN mice were higher at 4 h (Figure 14).

Compared with the normal group, glycyrrhetinic acid levels were higher in the heart, spleens, and lungs of mice with DN at 30 min. DNs had higher levels of glycyrrhetinic acid in their hearts and kidneys at 4 h. Levels of glycyrrhetinic acid in the hearts, kidneys, and intestines of mice with DN were higher at 8 h (Figure 15).

## 4 Discussion

In this study, we used 12-week-old db/db mice as a diabetic nephropathy mouse model and 12-week-old db/m mice as a normal comparison group. The db/db mouse was a mutant type, with the line of leptin receptor gene defect picked from C57BL/6J mice by the Jackson Laboratory of the United States. The db/db mouse spontaneously develops type 2 diabetes and is similar to clinical type 2 diabetes. db/db mice display obesity, hyperlipidemia, and hyperglycemia after 4 weeks of age. Diabetic nephropathy is found after 8–12 weeks of age (Gerald et al., 2013; Ponchiardi et al., 2013). The experimental results showed that the FBG, Scr, BUN, TC, TG, and 24 h U-Alb of 12-week db/db mice were significantly higher than those of the normal control group (*p <* 0.05). The kidneys of 12-week db/db mice also showed obvious glomerular lesions.

Some researchers have investigated *in vivo* content analysis methods for Huangqi Liuyi decoction (Zeng et al., 2018), but a method for simultaneously quantifying various ingredients in multiple organs has not yet been reported. In this research, HPLC-MS/MS was used to analyze the tissue distribution of the six active ingredients of HQD in normal and DN mice. Furthermore, the methodology, including specificity, linearity, accuracy, precision, extraction efficiency, matrix effect, and stability, was verified. The content of drugs in tissues and organs is usually difficult to measure, in part because there are many factors that interfere with the process. Triple quadrupole mass spectrometry is often used to determine trace components in biological samples, such as blood, urine, and tissue, in pharmacokinetic studies. In its multi-reaction monitoring scanning mode, the precursor ions of the tested component are prescreened in the first quadrupole (Q1), separated in the second quadrupole (Q2), and identified and quantified in the third quadrupole (Q3). In comparison with the traditional triple quadrupole mass spectrometry approach, the QTRAP® LC-MS/MS system (Sciex, Framingham, MA, United States) is equipped with an additional linear ion trap at the third quadrupole (Q3). This new generation of HPLC-MS/MS system, which is equipped with a unique scanning mode (incorporating multi-reaction ion monitoring, information-dependent acquisition, and enhanced ion scanning), can obtain the most abundant information of the tested components in a single operation, including ion pair retention time, relative peak strength, and mass spectrum. This greatly improves the detection sensitivity. After oral administration of HQD, six ingredients in various tissues of mice were determined by triple quadrupole linear ion trap mass spectrometry. At the same time, the precipitation of protein in mouse tissues by protein precipitants, such as methanol, acetonitrile, and ethyl acetate, was investigated. When methanol was used as a protein precipitant, the sample preparation was time-saving and stable, and the six active ingredients in the measured HQD were least disturbed by endogenous substances. As a consequence, methanol was selected as the precipitation solvent for sample pretreatment. Through methodological investigation, it was found that the established analytical methods of various components in mouse tissue samples met the requirements of biological sample determination.

The phenomenon by which a drug is transported between blood and tissues is called distribution. In oral administration, the drug is first absorbed into blood and then distributed to various tissues and cells throughout the body. The distribution process is usually completed quickly (Chen and Li, 2013). If the main tissue to which the drug is distributed happens to be the drug’s site of action, there is a close relationship between drug distribution and efficacy. Drug distribution to a non-active site is often closely related to drug toxicity and accumulation of the drug in the body. Therefore, investigating the *in vivo* distribution characteristics of drugs is useful in forecasting its pharmacological effects as well as the degree of *in vivo* retention and toxic effects (Wei and Zhang, 2004; Hu et al., 2020). In this study, the anti-DN effect of HQD was determined. After oral administration of HQD, six ingredients (astragaloside IV, calycosin-7-O-β-glucoside, calycosin glucuronide, ononin, formononetin, and glycyrrhizic acid) were detected in murine tissues regardless of the presence of diabetes. These six active ingredients were rapidly and widely distributed in multiple organs, albeit mainly in the lungs, kidneys, liver, spleen, and heart, and especially in the kidneys and lungs. Confirmation of the greatest distribution occurring in lung and kidney tissue samples indicates that HQD distribution is targeted at the lungs and kidneys, demonstrating positive significance for the prevention and treatment of nephropathy.

The major audience of drugs are diseased patients. Whether or not the body is in a pathological state has important measures of impact on the pharmacokinetics of the drug. In comparison with the normal condition, the distribution of these six ingredients in the lung, kidney, spleen, and heart increased in mice with DN, and especially in the kidney. The increase in the distribution of kidneys under pathological conditions can effectively guarantee the development of a prescription effect. In short, tissue distribution of the six index ingredients of HQD in the DN model was quite different from that in normal mice, but the reasons for this are still unclear. The concentrations of six active ingredients in the blood can affect their distribution in the tissues. Moreover, the excretion of drugs in the kidneys mainly includes glomerular filtration and renal tubular secretion and reabsorption. Glomerulonephritis and renal tubular lesions in DN mice are obvious compared to those in normal mice (Rick et al., 1987; Kudo et al., 2009; Liu, 2016). Diabetes and renal insufficiency in these mice will lead to a reduction in plasma protein content. As a consequence, the free drug concentration will increase, making more of the drug penetrate into tissues. At the same time, the permeability of blood vessels may change under different pathological conditions, which makes it easier for drugs to penetrate into tissues through capillary vessels, resulting in a change in the drug concentration accordingly. Therefore, the different characteristics of the distribution of six active ingredients may have been caused by the pathological state. However, the underlying mechanism of these differences remains to be further studied. In subsequent studies, we will further explore the mechanism by which the pathological state of diabetic nephropathy changes the tissue distribution of the six active ingredients in HQD.

## 5 Conclusion

HQD could effectively improve diabetes nephropathy. Moreover, a rapid, reliable, and sensitive HPLC/MS-MS method for simultaneous determination of six ingredients in the tissues of DN and normal mice after oral administration of HQD was established and validated. The tissue distribution characteristics of six ingredients in normal and DN model mice after oral administration of HQD were significantly different, but the overall trend was similar. The results might provide a reference for further research on HQD.

## Data Availability

The original contributions presented in the study are included in the article/supplementary material, further inquiries can be directed to the corresponding author.
